# Pharmacodynamics of Dracorhodin Perchlorate and Its Inflammation-Targeting Emulsion Gel for Wound Healing

**DOI:** 10.3390/gels8110712

**Published:** 2022-11-04

**Authors:** Xiaojie Wang, Xue Guo, Ran Yu, Mingxing Yue, Xingjuan Li, Bo Liu, Zhiquan Pan

**Affiliations:** 1 School of Chemistry and Environmental Engineering, Wuhan Institute of Technology, Wuhan 430205, China; 2 School of Biological Engineering, Beijing Polytechnic, Beijing 100176, China; 3 College of Chinese Medicine, Beijing University of Chinese Medicine, Beijing 102488, China; 4 Britton Chance Center for Biomedical Photonics at Wuhan National Laboratory for Optoelectronics-Hubei Bioinformatics & Molecular Imaging Key Laboratory, Department of Biomedical Engineering, College of Life Science and Technology, Huazhong University of Science and Technology, Wuhan 430074, China

**Keywords:** dracorhodin perchlorate, inflammation targeting, emulsion, gel, wound repair

## Abstract

The mechanism of dracorhodin perchlorate for the repair of rat skin wounds was investigated. In order to screen a more favorable drug delivery system for wound repair, the therapeutic effect of dracorhodin perchlorate inflammation-targeted emulsion gel was compared with that of non-targeted emulsion gel on rat wounds. Compared with non-targeted emulsion gels, inflammation-targeted emulsion gels had a better transdermal penetration and lower potentials (−51.6 mV and −17.1 mV, respectively). The recovery of the wound from the dracorhodin perchlorate inflammation targeted emulsion gel group was better than that of the dracorhodin perchlorate inflammation non-targeted emulsion gel group and the positive drug group. Compared with the no-target emulsion gel group, the bFGF expression on day 7 and the EGF expression on day 14 in the targeted emulsion group showed 45.5% and 49.9% improvement, respectively. Pathological tissue slices showed that the epidermis, dermis, and basal layer inflammatory cells in the inflammation-targeted emulsion gel group and non-targeted emulsion gel group were significantly reduced, the granulation tissue proliferation was obvious, and the inflammation-targeted emulsion gel group was more effective. The results proved that dracorhodin perchlorate had a repairing effect on rat skin wounds, and its mechanism might be related to the promotion of the expression of EGF and bFGF in tissues.

## 1. Introduction

*Dracaena draconis* (*Daemonorops draco* Bl.), also known as *Daemonorops draco*, is a traditional Chinese medicine commonly used to promote blood circulation which can also aid to maintain hemostasis. It has been widely used in the folk medicine and has exhibited promising efficacy in the treatment of fracture, trauma, inflammation, blood circulatory disorders and cancer [1,2,3,4,5,6]. A number of the clinical studies and animal experiments have confirmed the significant efficacy of *Dracaena draconis* and *Dracaena draconis*-containing myogenic drugs in promoting both acute and chronic wound repair such as burns and pressure ulcers, but the mechanism underlying these therapeutic effects remain unclear [7,8]. There are various chemical components present in *Dracaena draconis*, including esters, flavonoids, saponins and phenols. The 2015 edition of the Chinese Pharmacopoeia states that the quality standard of *Dracaena draconis* is primarily based on dracorhodin as a quantitative index, which is a flavonoid extracted from *Dracaena draconis*. Dracorhodin perchlorate can be found in about 1.6% of *Dracaena draconis*, which is significantly higher and active than other components present in *Dracaena draconis* [9]. Since dracorhodin is relatively unstable and easily reduced to a monomer, it is often found in the form of a specific salt, termed dracorhodin perchlorate, which can constitute a stable state for dracorhodin. Wu et al. reported that the blood-depleting hormone perchlorate could effectively promote fibroblast proliferation and collagen formation [10], thereby promoting wound healing, but its mechanism of action has not been well established.

Artificial drug delivery for promoting wound healing could reduce the incidence of wound infection and related diseases. Although direct administration was possible for some wounds of skin surface, an undifferentiated drug delivery strategy will greatly damage normal tissues and increase patient pain, which seriously hinders the clinical application of the drug. Hence, it was urgent to accurately concentrate drugs at the wound site to improve the curative effect and minimize the damage to normal tissues. Skin wounds were associated with a state of persistent inflammation, originating a specific microenvironment characterized by increased temperature, alkaline pH, elevated enzymatic activity, high levels of reactive oxygen species, and changed potential. Therefore, using the unique physicochemical properties of the wound microenvironment to rationally design a drug delivery system with targeting ability was an effective means to solve this problem.

**Scheme 1:**
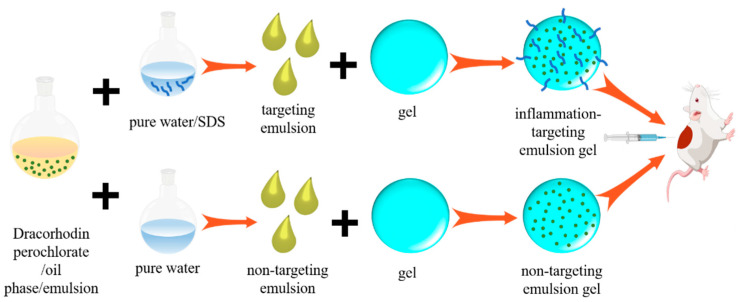
Schematic illustration of the preparation of dracorhodin perchlorate inflammation targeting/non-targeting emulsion gel.

A marked inflammatory response often occurs at the site of skin trauma, and an abnormally high expression of the cationic proteins such as transferrin at the site of inflammation can cause the site of inflammation to be positively charged [11]. It has been demonstrated that a reduction in inflammation could be achieved when the potential of the preparation was less than −30 mV [12]. In view of the above findings, in this work, a pilot study on the wound healing in rats by using an inflammation-targeting emulsion gel of hematinic perchlorate in comparison with a non-targeting emulsion gel was performed to explore the effects and mechanism of hematinic perchlorate in promoting the wound healing in rats, and to preliminary screen a suitable drug delivery system (Scheme 1). The findings can lead to the identification of a new wound-healing drug for clinical use, and a targeted preparation form of emulsion gel can be prepared based on it.

## 2. Results and Discussion

### 2.1. Potential Measurement

The zeta potential of three batches of inflammation-targeting emulsion was found to be −48.3 mV, −53.9 mV, and −52.6 mV respectively, with a mean of −51.6 mV. On the contrary, the zeta potential of three batches of non-targeting emulsion was −15.2 mV, −18.6 mV, and −17.5 mV respectively, with a mean of −17.1 mV.

### 2.2. Percutaneous Permeability

**Figure 1:**
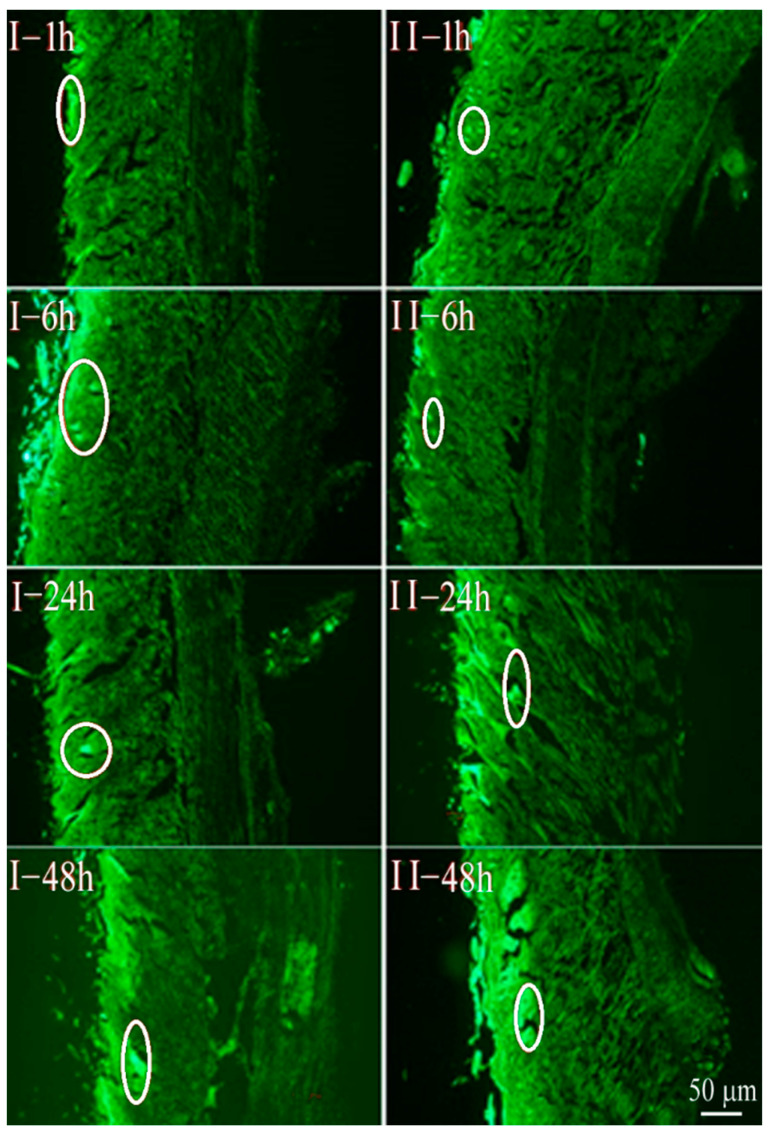
Fluorescence imaging of skin at different time after treated with dracorhodin perchlorate non-targeting emulsion gel (**left**) or dracorhodin perchlorate inflammation targeting emulsion gel (**right**). Scale bar is 50 μm.

The fluorescence intensities of the two administered groups increased significantly with time, which indicated that the amount of drug entering the skin increased proportionately with time. However, at the same time, the depth of the inflammation-targeting emulsion gel into the skin was observed to be deeper than that of the non-targeting emulsion gel (Figure 1), which might be possibly due to the faster generation of electrostatic adsorption between the inflammation-targeting emulsion gel and the positively charged inflammatory tissues.

### 2.3. Morphological Changes Observed in the Rats

**Figure 2:**
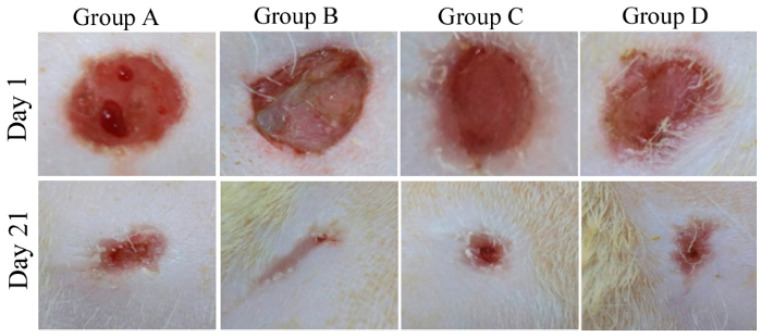
Pictures of wound after different treatments on day 1 and day 21. Notes: (A): model group; (B): dracorhodin perchlorate inflammation targeting emulsion gel group; (C): dracorhodin perchlorate non-targeting emulsion gel group; (D): positive group.

SD rats were randomly divided into four groups, namely (Group A) the model control group, (Group B) inflammation-targeting emulsion gel group, (Group C) non-targeting emulsion gel group, and (Group D) positive group (*n* = 10). The morphological changes of scalds after treated with dracorhodin perchlorate inflammation-targeting emulsion gel and dracorhodin perchlorate non-targeting emulsion gel were observed (Table 1). After 1 day of modeling, the epidermis of the wounds in rats of each group were hard, red and appeared to display a tendency to scab, but the wound area showed no substantial change. There were no significant differences between the groups (Figure 2).

After administration for 7 days, a few wounds became dehisced. The wound edge after dehiscence in-group A displayed yellow exudates and was overall dark red. On the contrary, the wounds in group B were pale red in color with a small amount of dark red exudate, and there was a smaller wound area with evidence of healing. Non-dehisced wounds in groups C and D were observed to be non-exudative, dark red, stiffer with upturned edges, and evidence of healing, but group C exhibited a more marked effect than group D.

After 14 days of administration, the wounds in group A were found to be red with dry scabs and exudates; Groups B and C healed well with smaller, reddish wounds, and there was no presence of exudate. The wounds in group D were reddish and healed with smoother skin than the wounds in group A, thus appearing pale white and less pigmented. Among all these, group B healed most prominently.

After 21 days of administration, the wounds of group A appeared to be light red with visible scars, but the wounds in groups B and C healed completely (Figure 2).

### 2.4. Observation of Pathological Sections

**Figure 3:**
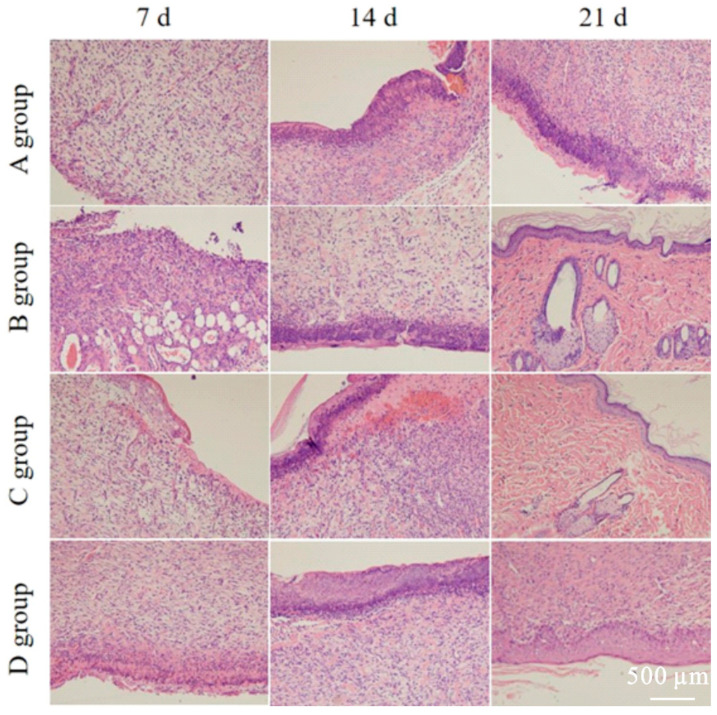
H&E staining results of traumatic tissue. Notes: (A): model group; (B): dracorhodin perchlorate inflammation targeting emulsion gel group; (C): dracorhodin perchlorate non-targeting emulsion gel group; (D): positive group. Scale bar is 500 μm.

Four different fields were selected and photographed under a 10× ocular lens to observe the histological changes of rat wounds. At 7 days, the epidermal layer of each group was found to be thickened. There was a large amount of inflammatory cell infiltration in the dermal layer in group A, but it was less in the administration groups. At day 14, group B displayed a homogeneous tissue arrangement, whereas groups C and D had decreased inflammatory cell infiltration and increased numbers of the fibroblasts. At day 21, epidermal layer structures had formed in group A, and the dermal layer still showed more inflammatory cell infiltration with a few fibroblasts. The neoplastic epidermal layer of group D had a more well-defined tissue structure with denser collagen fibers and a few inflammatory cell infiltrates in the interstitium. On the contrary, Groups B and C showed an intact epidermal layer. The specific results have been shown in Figure 3.

### 2.5. Expressions of bFGF and EGF

**Figure 4:**
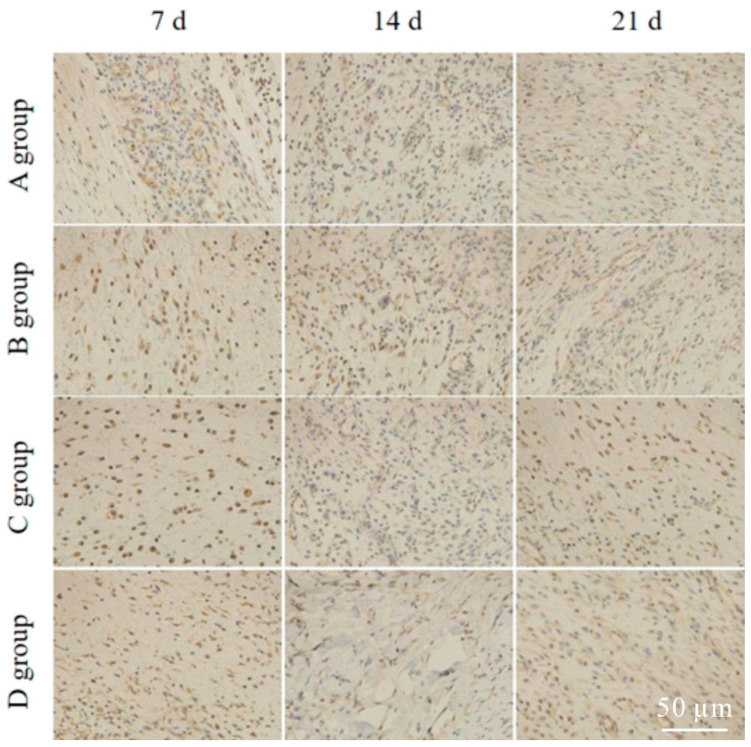
Expression results of bFGF at different time points. Notes: (A): model group; (B): dracorhodin perchlorate inflammation targeting emulsion gel group; (C): dracorhodin perchlorate non-targeting emulsion gel group; (D): positive group. Scale bar is 50 μm.

**Figure 5:**
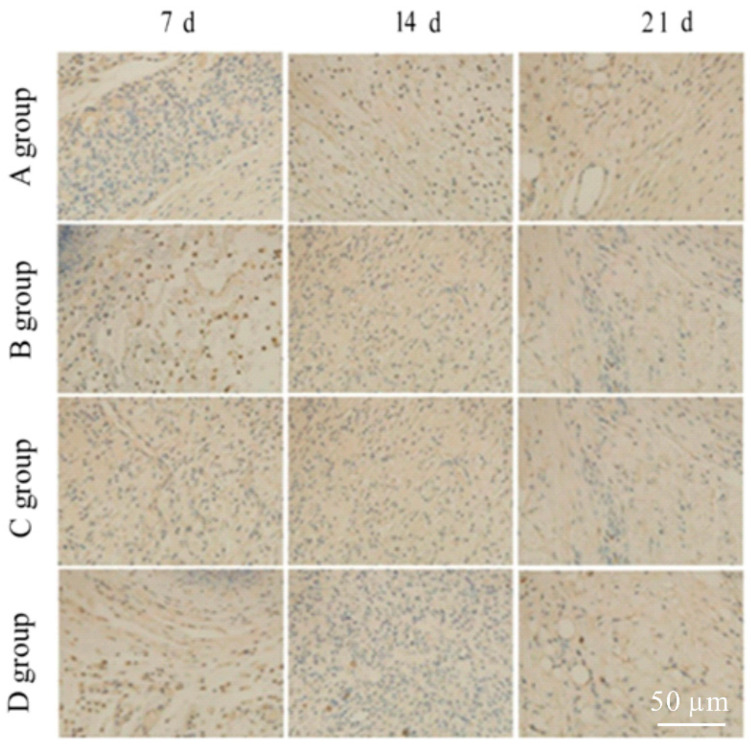
Expression results of EGF at different time points. Notes: (A): model group; (B): dracorhodin perchlorate inflammation targeting emulsion gel group; (C): dracorhodin perchlorate non-targeting emulsion gel group; (D): positive group. Scale bar is 50 μm.

The different sections were viewed under a microscope, and four fields were selected under a 40× ocular lens. The mean optical density values of the whole tissue pictures were measured using IPP-6.0 software. During the process of wound healing in rats of group B, the expression of bFGF in the granulation tissues was found to be significantly higher than that in the model group when administered at 7 d and 21 d (Figure 4). The expression of EGF was also significantly higher than that in the model group at 14 d and 21 d (Figure 5), with statistically significance difference when compared with the model group (* *p* < 0.05). The detailed results have been shown in Figure 4 and Figure 5 and Table 2 and Table 3.

### 2.6. Discussion

It has been described in Traditional Chinese Medicine that *Dracaena Draconis* can exhibit functions of dissipating stasis and relieving away saprophytic muscles. Da Ming Ben Cao in Chinese medicine states that it “treats all bad scabies those are long and bad”, so it often appears in all different kinds of myogenic formulas. According to statistics, *Dracaena Draconis* is the most frequently used medicine in the myogenic formula reported in various ancient medical literatures [13]. A number of the modern clinical studies have also confirmed that *Dracaena Draconis* and the myogenic drugs containing possess significant efficacy in promoting wound healing [7,8].

Dracorhodin perichlorate is the stable state of dracorhodin, and it is the main active component in *Dracaena Draconis*. Zhang et al. [14] demonstrated that the early formation of mature type I collagen could be effectively promoted by dracorhodin perichlorate in the range of 30–100 mg·mL^−1^, thereby resulting in an increased wound-healing quality. Qiao et al. [15] also primarily focused on examining the regulatory effects of dracorhodin perichlorate on collagen in the wound tissues while investigating the mechanisms of action of dracorhodin perichlorate on the wound-healing process in rats. Meanwhile, cellular active factors also play an extremely important role in the process of skin injury and repair.

In this paper, EGF and bFGF were primarily studied as the two key cytokines involved in the skin wound-healing process. As one of the most important mitogens in epidermal cells, EGF could stimulate epithelial cells and fibroblast proliferation and promote the filling of the granulation tissue. bFGF-expressing cells are the main functional cells in the wound healing. During skin healing, the expression of bFGF and EGF was found to be elevated. It was noted that comparing with the model group, the expressions of both EGF and bFGF were significantly increased after 21 days in both inflammation targeting and non-targeting emulsion gel groups (*p* < 0.05), whereas the expression of EGF was significantly increased after 14 days in inflammation, targeting the emulsion gel group (* *p* < 0.05). All these findings indicated that dracorhodin perchlorate could promote the expression of both EGF and bFGF in the wound tissues [16,17,18,19,20,21,22,23]. Moreover, inflammation-targeted emulsion gel was observed to be more suitable for use because of its potent inflammation targeting properties. Therefore, this study aimed to find the missing link in the related research and investigated the potential mechanism of dracorhodin perichlorate’s action on the various active cytokines in the wound tissues.

In recent years, percutaneous drug delivery systems have become one of the research hotspots in the field of pharmaceutics based on their unique superiority. Herein, dracorhodin perichlorate was prepared as inflammation-targeting and non-targeting emulsion gels for application as wound-healing treatment in rats. Both emulsion gels were easy to coat and displayed good stability, but they exhibited large differences in potential. In addition, previous reports have shown that emulsion gel demonstrated a wide range of advantages, including convenient mode of administration, avoidance of liver first-pass effect, reduced gastrointestinal irritation, prolonged drug action time, reduced frequency of administration, maintenance of steady-state blood concentration of drugs, and effectively improved patient medication compliance [24].

Our experimental results have clearly shown that both dracorhodin perchlorate non-targeting and inflammation-targeting emulsion gels could promote wound healing effectively, which might be attributed to the following two major aspects:

1. When the skin is damaged and infected, the wound will continually release different inflammatory mediators to cause an inflammatory response. However, at the site of inflammation, some cation-carrying proteins with aberrantly high expression will render the surface of inflamed tissues positively charged [11]. Based on this hypothesis, Jubeh et al. [25] prepared negatively charged liposomes of different zeta potential, and they could adsorb twice as strongly as the positively charged and electroneutral liposomes on inflammatory tissue sites in an in vitro adhesion assay in rats. Based on the specific charge characteristics of the inflammation site, the inflammation-targeting emulsion gel prepared in this study showed obvious targeting phenotypes due to its significantly stronger property of negative charge than the non-targeting emulsion gel.
2. When the skin is damaged and the wound develops, the normal epithelial potential undergoes a short circuit that can result in an outflow of current from the central site of the wound and a relatively stable current circuit at the wound edge. When the inflammation-targeting emulsion gel acts on a wound equivalent to an endogenous electric field, the preparation itself is equivalent to an exogenous electric field, under whose action the cells at the periphery of the wound can possibly move faster to promote the wound healing [26,27,28,29]. In this study, the potential of the inflammation-targeting emulsion gel differed greatly from the non-targeting one, being −51.6 mV and −17.1 mV, respectively. However, because wound tissue cells can migrate faster in a specific direction in response to the higher electric fields, the time required for the healing of skin lesions in rats was significantly shorter after administration of targeting emulsion gel compared with the non-targeting one.

## 3. Conclusions

In this study, we developed an inflammation-targeted dracorhodin perchlorate emulsion gel for wound repair. Compared with non-targeted emulsion gels, inflammation-targeted emulsion gels presented a better transdermal penetration. The results of wound recovery indicated that the dracorhodin perchlorate inflammation targeted emulsion gel group possessed better effect than that of the dracorhodin perchlorate inflammation non-targeted emulsion gel group and the positive drug group. Compared with the no-target emulsion gel group, the bFGF expression on day 7 and the EGF expression on day 14 in the targeted emulsion group showed 45.5% and 49.9% improvement, respectively. In addition, the expression of bFGF and EGF in the granulation tissues treated with inflammation targeted emulsion gel was found to be significantly higher than those treated with other methods. This work provides a promising insight for exploring dracorhodin perchlorate inflammation targeted emulsion gel for wound healing.

## 4. Materials and Methods

### 4.1. Animals

SD rats, half male and female, weighing 200–220 g were used. Qualification No was: SYXK (Beijing, China) 2020-0033. They were purchased from SPF (Beijing, China) Experimental Animal Technology Co., Ltd. and were raised in the animal room of Beijing University of Chinese Medicine.

### 4.2. Reagents and Chemicals

Glycerin monostearate was purchased from Shanghai Maclin Biochemical Technology Co., Ltd. (Shanghai, China). Soya bean lecithin was obtained from Aiweituo Pharmaceutical Technology Co., Ltd. (Shanghai, China). Jing Wan Hong ointment was purchased from Tianjin DarentangJingwanhong Pharmaceutical Co., Ltd. (Tianjin, China). Neutral formalin was obtained from Beijing Yili Fine Chemicals Co., Ltd. (Beijing, China). Epidermal growth factor (EGF) antibody, basic fibroblast growth factor (bFGF) antibody, and coumarin 6 were purchased from Shanghai Yuanye Bio-Technology Co., Ltd. (Shanghai, China). Chloralic hydras was obtained from the Tianjin Fuchen chemical reagent factory. Benzylpenicillin sodium for injection was purchased from Jiangxi Keda Animal Pharmaceutical Co., Ltd. (Fuzhou, China). Povidone iodine was obtained from Hunan Guangshengyuan Pharmaceutical Technology Co., Ltd. (Changsha, China). Veet depilatory cream was purchased from Reckitt Benckiser Household Products.

### 4.3. Instruments

The various instruments used in this study were as following: Microscopy (OLYMPUS BX51, Beijing, China, Beijing Ruichi Hengye Instrument Technology Co., Ltd.); Electronic analytical balance (Sartorius BS 110S, Beijing, China, Beijing Sartorius Scientific Instruments Co., Ltd.); Electric-heated thermostatic water bath (Tianjin, China, Tianjin Taisite Medical Equipment Inc.); Desktop high-speed centrifuge (3-18N, Changsha, Hunan Province, China, Hunan hengnuo Instrument Equipment Co., Ltd.); Ultrasonic cell disintegrator (Ningbo, Zhejiang Province, China, Ningbo Xinzhi Technology Co., Ltd.); Smart pet shavers (Handan, Hebei Province, China, Handan Fengfenghanzhuo Trading Co., Ltd.); Refrigerator (Beijing, China, TCL Home Appliances Group Co., Ltd.); Numerically controlled ultrasound (Kunshan, Jiangsu Province, China, KQ-3200DE, Kunshan Ultrasonic Instrument Co., Ltd.); Fluorescence inverted biomicroscope (Beijing, China, TS-2, Beijing Cnrico Technology Co., Ltd.); Electronic balance (Shanghai, China, Shanghai Hengping Apparatus & Instruments Factory); ZS90 Nanosizer (UK Malvern); Surgical instruments (e.g., gavage needle, syringe, forceps, hemostat, gauze, cotton pellet, cotton swab, tissue clipping).

### 4.4. Preparation of Emulsion Gel

#### 4.4.1. Preparation of Dracorhodin Perchlorate Inflammation-Targeting Emulsion Gel

Glycerin monostearate, soya bean lecithin and dracorhodin perchlorate (quality ratio 5:10:1) were mixed and sonicated to dissolve in ethanol. SDS was dissolved into pure water and slowly added to mixture solution under stirring to obtain an inflammation-targeting emulsion. After mixed proportionally with the gel, dracorhodin perchlorate inflammation-targeting emulsion gel was obtained.

#### 4.4.2. Preparation of Dracorhodin Perchlorate Non-Targeting Emulsion Gel

The preparation was performed based on the same procedure as described in the section of preparation of dracorhodin perchlorate inflammation-targeting emulsion gel, but no charge-imparting agent (SDS) was added.

### 4.5. Zeta Potential Measurement

Appropriate amounts of emulsions were pipetted finely and diluted to 100× with distilled water. Thereafter, emulsion dilution was slowly injected into the zeta potential cup after being drawn up by a 5 mL disposable syringe. After scrutinizing and emptying the bubbles, the zeta potentials of emulsion gels were measured with a ZS Nanosizer (Malvern, UK).

### 4.6. In Vivo Fluorescence Transdermal Experiments

#### 4.6.1. Preparation of Fluorescently Labeled Dracorhodin Perchlorate Inflammation-Targeting and Non-Targeting Emulsion Gels

The fluorescence labeled dracorhodin perchlorate inflammation-targeting and non-targeting emulsion gel were synthesized following the preparation process of dracorhodin perchlorate emulsion gel after adding 100 μL of 0.5% coumarin 6 solution.

#### 4.6.2. Preparation and Observation of Microsamples

The hair removal of the dorsal integuments of seven SD rats was accomplished using an electric shaver fitted with a depilatory cream. The dorsal preps of each rat were further differentiated into two different parts, and fluorescently labeled non-targeting emulsion gel (Group I) and dracorhodin perchlorate inflammation-targeting emulsion gel (Group II) were then administered separately using a fine glass rod coupled with a syringe. Each part was 1 cm × 1 cm in size. The rats were housed in rat cages covered in black plastic bags. After anesthetizing the rats with an intraperitoneal injection of chloral hydrate at 1, 6, 24, and 48 h after administration, all the different parts of the skin were removed, fixed with 10% formaldehyde, and subjected to a series of steps including dehydration, wax immersion, embedding, and sectioning to make longitudinal sections, which were kept frozen and protected from the light. The sections were thereafter mounted under a fluorescence inverted microscope (40×; excitation wavelength = 488 nm) for observation and image acquisition.

### 4.7. Grouping of the Test Animals, Model Preparation, and Administration Method

The model preparation and administration method were carried according to previous works [30,31]. Forty SD rats were randomly divided into the model control group (Group A), inflammation-targeting emulsion gel group (Group B), non-targeting emulsion gel group (Group C) and positive group (Group D). The various preparations were applied topically, once a day, at a dose of 0.2 g to form a 1 mm thick drug layer on the wound in each dosing group. The rats in the model group were conventionally raised without applying any drugs, and the disinfection with iodophor allowed the wounds to heal naturally. On test day 1, the backs of the rats were depilated using a razor knife combined with a depilatory cream and subsequently disinfected with alcohol. Two epidermal skin wounds with a diameter of 18 mm were cut at 1 cm each on the left and right sides of the dorsal spine of rats using the surgical scissors. Approximately 0.01 g of sodium penicillin powder for injection was subsequently spread on each wound, and the wound was then disinfected using iodophor. After the rats were awake after anesthesia, the routine raising was performed. The preparations are subsequently applied daily to the wound site, and daily application at the same time was guaranteed. The test cycle was fixed to 21 days.

### 4.8. Pharmacodynamic Indicators of Wound

#### 4.8.1. Macroscopic Observation of Wound

The direct observation was performed when the rats were resuscitated after the wound modeling. The wound status was observed on days 7, 14, and 21 after wounding, and exudates, infections, and scab redness were recorded.

#### 4.8.2. Wound Area

The wound was traced using transparent sulphate paper, and the wound area was taken as the original area after the wound modeling for 24 h. The unhealed area of each group was recorded after 7, 14, and 21 days.

#### 4.8.3. Wound-Healing Rate

The mass of transparent sulfuric acid paper was weighed with a balance to replace area by mass. Thereafter, the wound-healing rate was calculated according to the following formula: “wound-healing rate = [(original wound area − the residue wound area at each time point)/original wound area] × 100%” [32,33].

#### 4.8.4. Histopathological Observations

The wound skin tissues from the rats in each group at days 7, 14, and 21 were harvested and fixed in 10% neutral formalin solution for 48 h, after which HE-stained sections were generated for observation of the histopathological changes in the wound under a fluorescence inverted biomicroscope.

#### 4.8.5. Immunohistochemistry

The wound skin tissues from the rats at days 7, 14, and 21 in each group were dehydrated, transparent, macerated, embedded, sectioned, and processed for immunohistochemical sections. The expression of EGF and bFGF proteins was observed under a 40× light microscope. Specifically, four different fields of view were selected for each section, and the integrated optical density (IOD) values were calculated and averaged over the blue region using IPP-6.0 Image analysis software. The study was conducted in accordance with the Declaration of Helsinki, and the protocol was approved by the Ethics Committee of Beijing University of Traditional Chinese Medicine (No.: BUCM-4-2016011003-1003).

## Figures and Tables

**Table 1:** Wound-healing rate at each time point of scald (x ± s).

Group	Wound-Healing Rate			
	1 d	7 d	14 d	21 d
A	0.13 ± 0.09	0.43 ± 0.15	0.71 ± 0.20	0.82 ± 0.21
B	0.16 ± 0.10	0.66 ± 0.15	0.69 ± 0.19	0.9 ± 0.18
C	0.14 ± 0.12	0.64 ± 0.11	0.75 ± 0.16	0.87 ± 0.18
D	0.15 ± 0.13	0.65 ± 0.14	0.77 ± 0.15	0.88 ± 0.14

A: model group, B: dracorhodin perchlorate inflammation-targeting emulsion gel group, C: dracorhodin perchlorate non-targeting emulsion gel group, D: positive group.

**Table 2:** Expression of bFGF in the granulation tissue of rats at the different stages (x ± s).

Group	Expression of bFGF (pg/mL)		
	7 d	14 d	21 d
A	13.19 ± 3.15	15.51 ± 6.83	7.42 ± 1.91
B	24.34 ± 5.96 *	9.84 ± 1.19 ^a^	15.05 ± 4.71 *
C	16.73 ± 2.03	15.10 ± 3.30	16.18 ± 9.80
D	20.53 ± 9.79	8.34 ± 2.50	12.96 ± 3.39 *

A: model group, B: dracorhodin perchlorate inflammation-targeting emulsion gel group, C: dracorhodin perchlorate non-targeting emulsion gel group, D: positive group, * *p* < 0.05 compared with the model group. ^a^, *p* < 0.05 compared with C group.

**Table 3:** Expression of EGF in the granulation tissue of rats at the different stages (x ± s).

Group	Expression of EGF (pg/mL)		
	7 d	14 d	21 d
A	6.77 ± 1.02	5.39 ± 1.10	9.21 ± 0.76
B	8.96 ± 2.49	13.78 ± 5.30 *	17.40 ± 5.55 *
C	6.90 ± 1.95	9.19 ± 5.34	14.23 ± 2.63
D	9.62 ± 2.54	8.71 ± 2.30	11.39 ± 3.23

A: model group, B: dracorhodin perchlorate inflammation-targeting emulsion gel group, C: dracorhodin perchlorate non-targeting emulsion gel group, D: positive group, * *p* < 0.05 compared with the model group.

## Data Availability

Not applicable.
